# Regulation of P450 oxidoreductase by gonadotropins in rat ovary and its effect on estrogen production

**DOI:** 10.1186/1477-7827-6-62

**Published:** 2008-12-16

**Authors:** Yoshihiko Inaoka, Takashi Yazawa, Tetsuya Mizutani, Koichi Kokame, Kenji Kangawa, Miki Uesaka, Akihiro Umezawa, Kaoru Miyamoto

**Affiliations:** 1 Department of Biochemistry, Faculty of Medical Sciences, University of Fukui, Fukui 910-1193, Japan; 2 National Cardiovascular Research Center, Osaka 565-8565, Japan; 3 National Research Institute for Child Health and Development, Tokyo 157-8535, Japan

## Abstract

**Background:**

P450 oxidoreductase (POR) catalyzes electron transfer to microsomal P450 enzymes. Its deficiency causes Antley-Bixler syndrome (ABS), and about half the patients with ABS have ambiguous genitalia and/or impaired steroidogenesis. POR mRNA expression is up-regulated when mesenchymal stem cells (MSCs) differentiate into steroidogenic cells, suggesting that the regulation of POR gene expression is important for steroidogenesis. In this context we examined the regulation of POR expression in ovarian granulosa cells by gonadotropins, and its possible role in steroidogenesis.

**Methods:**

Changes in gene expression in MSCs during differentiation into steroidogenic cells were examined by DNA microarray analysis. Changes in mRNA and protein expression of POR in the rat ovary or in granulosa cells induced by gonadotropin treatment were examined by reverse transcription-polymerase chain reaction and western blotting. Effects of transient expression of wild-type or mutant (R457H or V492E) POR proteins on the production of estrone in COS-7 cells were examined in vitro. Effects of POR knockdown were also examined in estrogen producing cell-line, KGN cells.

**Results:**

POR mRNA was induced in MSCs following transduction with the SF-1 retrovirus, and was further increased by cAMP treatment. Expression of POR mRNA, as well as Cyp19 mRNA, in the rat ovary were induced by equine chorionic gonadotropin and human chorionic gonadotropin. POR mRNA and protein were also induced by follicle stimulating hormone in primary cultured rat granulosa cells, and the induction pattern was similar to that for aromatase. Transient expression of POR in COS-7 cells, which expressed a constant amount of aromatase protein, greatly increased the rate of conversion of androstenedione to estrone, in a dose-dependent manner. The expression of mutant POR proteins (R457H or V492E), such as those found in ABS patients, had much less effect on aromatase activity than expression of wild-type POR proteins. Knockdown of endogenous POR protein in KGN human granulosa cells led to reduced estrone production, indicating that endogenous POR affected aromatase activity.

**Conclusion:**

We demonstrated that the expression of POR, together with that of aromatase, was regulated by gonadotropins, and that its induction could up-regulate aromatase activity in the ovary, resulting in a coordinated increase in estrogen production.

## Background

The ovary is one of the major steroidogenic organs, and ovarian follicular development is initiated by follicle stimulating hormone (FSH) secreted by the pituitary gland. FSH-induced follicular development involves granulosa cell proliferation and differentiation [1,2]. FSH induces steroidogenic enzymes in ovarian granulosa cells leading to the production of steroid hormones, including estrogens. P450 aromatase (CYP19) is one of the steroidogenic enzymes regulated by FSH, and catalyzes the biosynthesis of estrogens from C19 steroids in the granulosa cells [3].

P450 oxidoreductase (POR) is an 82-kDa membrane-bound flavoprotein [4,5] responsible for the transfer of electrons from nicotine adenine dinucleotide phosphate to flavin adenine dinucleotide (FAD) and flavin mononucleotide (FMN), and finally to microsomal P450 enzymes, including aromatase, as well as to several non-P450 enzymes [6,7]. Several clinical studies have reported that mutations in POR are sufficient to cause Antley-Bixler syndrome (ABS), which is characterized by skeletal malformations. In some cases, ABS patients have genital ambiguity, and in some of the cases female ABS patients present with polycystic ovary syndrome [6,8-11]. The clinical features are primarily explained by the impaired activities of CYP21A2, CYP17A1, and CYP19 involved in steroidogenesis [11,12].

Bone marrow-derived mesenchymal stem cells (MSCs) are defined as pluripotent cells, and have been shown to differentiate into adipocytes, chondrocytes, osteoblasts, and hematopoietic-supporting stroma, both in vivo and ex vivo [13-15]. We recently reported that stable transfection of MSCs with the transcription factor, steroidogenic factor-1 (SF-1), followed by treatment with cAMP, led to their conversion into steroidogenic cells [16]. These cells offer a possible clinical source of stem cells for the treatment of diseases of steroidogenic organs, and represent a powerful tool for studying the differentiation of the steroidogenic lineage.

In this study, we investigated the changes in gene expression in MSCs during differentiation into steroidogenic cells. POR gene expression, as well as that of various steroidogenic enzymes, was induced by SF-1 and cAMP. It was also induced by gonadotropins in rat ovary and granulosa cells. We also examined the effects of wild-type and mutant POR expression on aromatase activity, and the effects of knockdown of endogenous POR in mammalian cell lines. Our results suggest that up-regulation of POR expression by gonadotropins, as well as that of aromatase, could enhance ovarian estrogen synthesis.

## Methods

Diethylstilbestrol (DES), 8-bromoadenosine 3'-5'-cyclic monophosphate (8-Br-cAMP), and gentamicin were purchased from Sigma (St. Louis, MO). Ovine FSH was obtained from the National Institutes of Health (NIH). Equine chorionic gonadotropin (eCG) was purchased from Teikokuzouki, Inc. (Tokyo, Japan). Human chorionic gonadotropin (hCG) was obtained from Sankyo (Tokyo, Japan). Ex Taq DNA polymerase, *Escherichia coli *DNA ligase, and DNA ligation kit were purchased from Takara Biomedicals (Kyoto, Japan). Ham's F-12/DMEM and DMEM were purchased from Wako Chemicals (Tokyo, Japan). Reagents for real-time polymerase chain reaction (PCR) were purchased from Applied Biosystems (Foster, CA). HiPerFect Transfection Reagent and fluorescently labeled high-performance purity grade small interfering RNA (siRNA), used for transfection control, were purchased from QIAGEN (Valencia, CA). The QuikChange site-directed mutagenesis kit and Pfu DNA polymerase were purchased from Stratagene (La Jolla, CA). Genopure Plasmid Maxi Kit was purchased from Roche Applied Science (Penzberg, Germany). The Trizol reagent, Superscript III reverse transcriptase, *E. coli *DNA polymerase, RNase H, Lipofectamine Plus, pcDNA3 and pEF-1/V5-His A vectors were purchased from Invitrogen (Carlsbad, CA). A Protein Assay Kit was purchased from Bio-Rad Laboratories, Inc. (Hercules, CA). Anti-POR (sc-25270) and anti-glyceraldehyde-3-phosphate dehydrogenase (GAPDH) (sc-32233) antibodies were purchased from Santa Cruz Biotechnology, Inc. (Santa Cruz, CA). Anti-aromatase (MCA2077S) antibody was purchased from AbD Serotec (Oxford, UK). The enzyme immunoassay (EIA) kits for estrone and estradiol were purchased from Cayman Chemical (Ann Arbor, MI). SiRNAs for human POR, named POR #1 (Hs_POR-2, SASI_Hs01_00216914) and POR #2 (Hs_POR-3, SASI_Hs01_00216915), were purchased from Sigma-Aldrich Japan (Tokyo, Japan). pQCXIP retroviral vector kit was purchased from Clontech Laboratories (Palo Alto, CA).

### Microarray analysis

UE7T-13 MSCs [17], transduced or untransduced with SF-1 by retrovirus-mediated transfection, were cultured in DMEM supplemented with 50 mg/l gentamicin sulfate and 10% fetal bovine serum (FBS) in a humidified atmosphere containing 5% CO2 in 95% air at 37°C. Total RNA was isolated with Trizol reagent and Oligotex. cDNA was synthesized from total RNA using a Superscript III kit with T7-(dT) 24-mer primer harboring a T7 RNA polymerase promoter. The cRNA was then prepared using an Affymetrix GeneChip expression 3' amplification kit. Labeled cRNA was fragmented by incubation at 94°C for 35 min. Hybridization of cRNA for human U133 Plus 2.0 Affymetrix GeneChip and its analysis were performed as described previously [18].

### Rat granulosa cell culture

Granulosa cells were obtained from immature, SLC: SD female rats (21 days old) that had received injections of 2 mg DES in 0.2 ml sesame oil once daily for 4 d. The ovaries were then excised and granulosa cells were released by puncturing the follicles with a 26-gauge needle. Animals were treated according to NIH guidelines and the experiments were approved by the animal experiment committee of the University of Fukui. Granulosa cells were washed and collected by brief centrifugation, and cell viability was determined by trypan blue exclusion. The granulosa cells were then cultured in Ham's F-12/DMEM supplemented with 50 mg/l gentamicin sulfate and 0.1% bovine serum albumin on collagen-coated plates in a humidified atmosphere containing 5% CO_2 _in 95% air at 37°C [19].

### Hormone treatments and analysis of gene expression

Granulosa cells (5 × 10^6 ^cells) were cultured in 60-mm dishes in 5 ml medium. Ovine FSH (30 ng/ml) was added to the medium after 24 h of cell culture. The cultures were stopped at various time intervals. For the in vivo study, 21-day-old rats were primed with 30 IU eCG. After 48 h, the rats were primed with 30 IU hCG and ovaries were removed at various time intervals. KGN cells (kindly gifted by Dr. Toshihiko Yanase, Kyushu University, Fukuoka, Japan) were cultured in 60-mm dishes containing 7.5 × 10^6 ^cells in 5 ml medium and 8-Br-cAMP (final concentration 1 mM) was added to the medium 24 h after plating. The cultures were stopped at various time intervals. Total RNA was extracted from the cultured cells using Trizol reagent. Reverse transcription-PCR (RT-PCR) was performed as described previously [20]. The reaction mixture was subjected to electrophoresis in a 1.5% agarose gel, and the resulting bands were visualized by staining with ethidium bromide. The primer pairs used were: forward primer, 5'-caccacacctgtcatcatgg-3' (nucleotides 1612–1631) and reverse primer, 5'-tgaggtcggcagaagttagg-3' (nucleotides 2115–2134), for rat POR, product size: 523 bp (NM_031576); forward primer, 5'-ctggacgaaagttctattg-3' (nucleotides 679–698) and reverse primer 5'-gaagcaacatgacgtacaga-3' (nucleotides 1000–1019), for Cyp19, product size: 341 bp (NM_017085); and forward primer, 5'-ggacttctacgactggctgcagg-3' (nucleotides 541–563) and reverse primer, 5'-tcttgttggactcctcatccagg-3' (nucleotides 1141–1163), for hPOR, product size: 623 bp (NM_000941). The primer pairs for CYP19 (NM_000103), GAPDH (NM_002046), and beta-actin (NM_001101.3) have been described previously [16]. Real-time PCR was performed as described by Rutledge and Cote [21]. The primer pairs used were: forward primer, 5'-ggagacgctgctgtactacg-3' (nucleotides 1765–1784) and reverse primer, 5'-gctggacgtagaccttgtgg-3' (nucleotides 1891–1912), for human POR, product size 146 bp; forward primer, 5'-ggacttcgagcaagagatgg-3' (nucleotides 747–766) and reverse primer, 5'-aaggaaggctggaagagtgc-3' (nucleotides 862–881), for human beta-actin, product size 135 bp; forward primer, 5'-gatgacgggaacttggaaga-3' (nucleotides 662–681) and reverse primer, 5'-agctcatactggcgaatgct-3' (nucleotides 761–780), for rat POR, product size 119 bp; forward primer, 5'-tcctcagcagagaaactggaaga-3' (nucleotides 857–879) and reverse primer, 5'-cgtacagagtgacggacatggt-3', (nucleotides 986–1007) for cyp19, product size 151 bp; and forward primer, 5'-ggcgacctggaagtccaact-3' (nucleotides 47–66) and reverse primer, 5'-ggatctgctgcatctgcttg-3' (nucleotides 140–159), for rat 36B4, product size: 113 bp (NM_022402).

### Western blot analysis

In order to determine protein expression levels, granulosa, KGN, and COS-7 cells (purchased from American type culture collection) were washed twice in ice-cold phosphate-buffered saline and lysed in RIPA buffer (50 mM Tris-HCl (pH 8.0) containing 1% Nonidet P-40, 0.1% sodium dodecyl sulfate (SDS), 0.5% deoxycholate, and 1 mM phenylmethylsulfonyl fluoride). The cell debris was removed by centrifugation at 15,000 × g at 4°C for 15 min, and supernatants were used as cell lysates. Proteins were quantified using a Bio-Rad Protein Assay kit. Equal amounts of protein (30 μg) were resolved by SDS-polyacrylamide gel electrophoresis and transferred onto polyvinylidene difluoride membranes. Western blot analysis of POR, CYP19, and GAPDH was performed with antisera directed against POR (raised against amino acids 1–300 of human POR), CYP19 (synthetic peptide corresponding to amino acids 376–390 of human CYP19), and GAPDH, respectively. All antibodies were capable of reacting with both human and rat antigens, respectively. Western blot analysis of POR, aromatase, and GAPDH was performed with antisera directed against POR, CYP19, and GAPDH, respectively. Enhanced chemiluminescence western blot reagents (Amersham Pharmacia Biotech, Arlington Heights, IL) were used for detection.

### Plasmid constructs

Entire coding regions of human and rat POR were generated by RT-PCR using cDNA from UE7T-13 cells stably transfected with SF-1, and granulosa cells, respectively. The coding regions were then subcloned into the expression vector, pcDNA3. Human CYP19 was generated by RT-PCR using cDNA from KGN cells stimulated with 8-Br-cAMP for 12 h, and subcloned into pEF-1. Primer pairs used were: 5'-gagaGAATTCgccaccATGGATTACAAGGATGACGACGATAAGggagactcccacgtgga-3' and 5'-gagaCTCGAGctagctccacacgtccaggg-3', for human POR; 5'-gagaGAATTCgccaccATGGATTACAAGGATGACGACGATAAGggggactctcacgaagac-3' and 5'-gagaCTCGAGctagctccacacatctagtga-3', for rat POR; and 5'-gagaGATATCgccaccATGGATTACAAGGATGACGACGATAAGgttttggaaatgctgaac-3' and 5'-gagaGCGGCCGCctagtgttccagacacctgtc-3', for human CYP19. In order to obtain mutant human PORs (R457H and V492E), site-directed mutagenesis of the human POR was performed using the QuikChange Site-Directed Mutagenesis Kit. Primer pairs used were: 5'-gccgcgcctgcaggcccActactactccatcgcct-3' and 5'-aggcgatggagtagtagTgggcctgcaggcgcggc-3', for R457H; and 5'-ccgcatcaacaagggcgAggccaccaactggctgc-3' and 5'-gcagccagttggtggccTcgcccttgttgatgcgg-3', for V492E.

### Transfection

COS-7 cells were cultured in DMEM supplemented with antibiotics and 10% FBS in a humidified atmosphere containing 5% CO_2 _and 95% air at 37°C. DNA transfections were carried out using Lipofectamine Plus reagent. All plasmids used for transfections were prepared using a Genopure Plasmid Maxi Kit. Cells (4 × 10^5^) were cultured in 60-mm dishes in 5 ml medium on the day before transfection. Three hours after co-transfection of human aromatase (1 μg/well) and rat POR (20, 50, or 100 ng/well) or pcDNA3, medium was changed to growth medium. After 48 h, the growth medium was changed to medium containing androstenedione (final concentration 10^-5 ^M). Culture medium was collected at various time points in order to observe the conversion of androstenedione to estrone.

### EIA

The levels of estrone in the media were measured using an EIA assay kit (Cayman) according to the manufacturer's instructions. Briefly, 50 μl of diluted medium (1:1000), estrone tracer, and antiserum were added to each well. They were incubated at room temperature for 2 h and developed with Ellman's reagent solution. The developed plates were measured using a plate reader at 405 nm.

### SiRNA transfection

KGN cells were cultured in DMEM/Ham's F12 supplemented with antibiotics and 10% FBS in a humidified atmosphere containing 5% CO_2 _and 95% air at 37°C. Knockdown of endogenous POR was carried out using siRNAs and HiPerFect reagent. Twenty-four hours after transfection, the culture medium was changed to DMEM/Ham's F12 supplemented with antibiotics and 10% FBS containing 1 mM 8-Br-cAMP and 10 μM androstenedione. Cells and medium were collected 48 h after transfection. SiRNAs for POR were POR #1 (sense: 5' GCACAUCUGUGCGGUGGUUTT, anti sense: 5' AACCACCGCACAGAUGUGCTT) and POR #2 (sense: 5' CCAACUGGCUGCGGGCCAATT, anti sense: UUGGCCCGCAGCCAGUUGGTT). They were targeted to the region approximately 1496 and 1568 nucleotides downstream from the beginning of the human POR (Ref seq NM_000941), respectively.

### Statistics

Data from each experiment were analyzed using the Student's t test. P < 0.01 was considered statistically significant.

## Results

### Changes in gene expression in human MSCs during differentiation into steroidogenic cells

**Figure 1:**
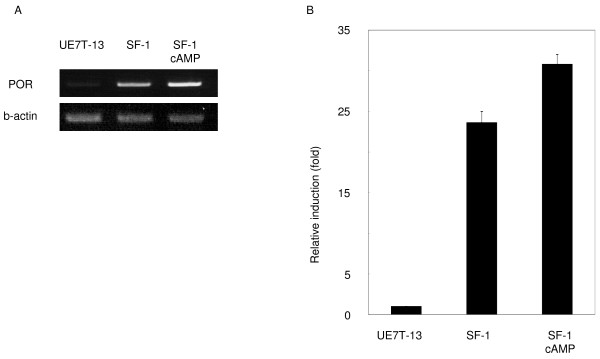
**Induction of POR mRNA in human MSCs**. A: RT-PCR analysis of POR mRNA in UE7T-13 cells transduced with SF-1 or pQCXIP (control) virus and cultured with or without 8-Br-cAMP for 2 d. B: Results of real-time PCR are also shown. Data are the mean ± SEM values of duplicate experiments.

In previous studies, we converted MSCs into steroidogenic cell-lineages such as Leydig cells and adrenocortical cells by expression of SF-1 and treatment with cAMP [16]. DNA microarray analysis was performed using control and SF-1-transduced human MSCs in order to examine alterations of gene expression. Using more than 22,000 probe sets (18,400 transcripts and variants, including 14,500 well-characterized human genes), a number of differences in gene expression levels between control cells and UE7T-13 cells stably expressing SF-1 were detected: expression levels of about 1,700 genes, including POR, were found to be increased more than 5-fold in SF-1- transduced cells (representative data from two independent experiments are shown in Table 1). Gene expression of StAR [22], CYP11A1 [23], HSD3B2 [24], CYP19 [3], and CYP17A1 [25], whose transcriptions are known to be regulated by SF-1, were induced. Consistent with the results of DNA microarray, RT-PCR and real-time PCR analysis showed that POR mRNA was markedly increased more than 20-fold in UE7T-13 cells transduced with SF-1. It was further induced by the addition of 8-Br-cAMP, and reached to about 31-fold (Fig. 1A and 1B). These results indicate that, concomitant with microsomal P450 genes, the POR gene was induced during the differentiation of MSCs into steroidogenic cells, and suggest that its up-regulation may contribute to the development of the steroidogenic phenotype.

**Table 1:** Subset of genes induced by SF-1 in UE7T-13 human mesenchymal stem cells.

Affymetrix ID	Ratio	Accession no.	Gene Symbol
211506_s_at	488.85	AF043337	IL8
201262_s_at	463.31	BC002416	BGN
204309_at	362.16	NM_000781	CYP11A1
202411_at	274.98	NM_005532	IFI27
206294_at	225.3	NM_000198	HSD3B2
202036_s_at	160.06	AF017987	SFRP1
204548_at	153.41	NM_000349	STAR
220187_at	144.86	NM_024636	STEAP4
205498_at	96.03	NM_000163	GHR
202037_s_at	94.77	AF017987	SFRP1
203680_at	40.13	NM_002736	PRKAR2B
203632_s_at	40.06	NM_016235	GPRC5B
203475_at	32.4	NM_000103	CYP19A1
204415_at	31.5	NM_022873	IFI6
203903_s_at	24.23	NM_014799	HEPH
201010_s_at	20.95	NM_006472	TXNIP
213006_at	20.92	AV655640	CEBPD
201009_s_at	18.38	NM_006472	TXNIP
205027_s_at	17.9	NM_005204	MAP3K8
205635_at	15.66	NM_003947	KALRN
205502_at	14.89	NM_000102	CYP17A1
201981_at	14.76	AA148534	PAPPA
201416_at	14.15	NM_003107	SOX4
205302_at	13.77	NM_000596	IGFBP1
215233_at	11.12	AA351360	PTDSR
216117_at	10.42	AK025114	EXOSC2
203148_s_at	10.34	NM_014788	TRIM14
205404_at	8.66	NM_005525	HSD11B1
209540_at	8.52	M29644	IGF1
205676_at	8.51	NM_000785	CYP27B1
207135_at	8.41	NM_000621	HTR2A
210002_at	8.36	D87811	GATA6
204135_at	8.31	NM_014890	DOC1
208928_at	8.18	AF258341	POR
212095_s_at	7.41	BE552421	MTUS1
216248_s_at	6.24	S77154	NR4A2
206645_s_at	5.29	NM_000475	NR0B1

### Induction of POR expression by gonadotropins in the ovary

**Figure 2:**
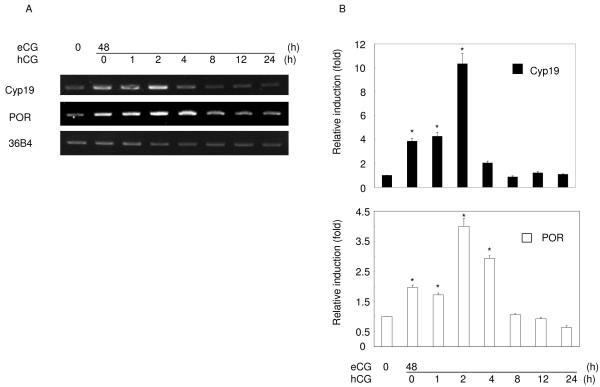
**Time-dependent changes in POR and aromatase mRNA expression in immature rat ovaries treated with eCG and hCG**. A: Expression of each mRNA was examined by RT-PCR. Immature rat ovaries were primed with 30 IU eCG for 48 h, followed with treatment with 30 IU hCG for the indicated times. B: Results of real-time PCR are also shown. Data are the mean ± SEM values of at least three independent experiments. *, *P *< 0.01.

In order to evaluate the relevance of above hypothesis, we examined the expression of POR in the ovary and in granulosa cells, an organ and cells that are well known to be involved in steroidogenesis or in the differentiation into steroidogenic by gonadotropins. Ovarian POR mRNA was detected at relatively low levels before gonadotropin treatment. It was induced after 48 h of eCG treatment, and was further increased within 2 h of the following hCG treatment (Fig. 2A and 2B). Its expression then fell substantially at 8 h, and continued to decline thereafter. The pattern of POR expression induced by eCG and hCG was similar to that of Cyp19 expression.

**Figure 3:**
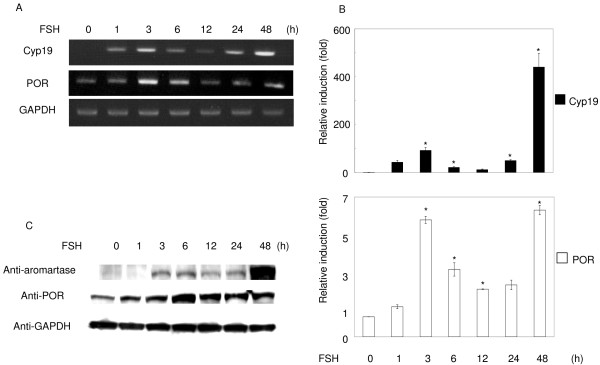
**Regulation of POR and aromatase mRNA and protein levels by FSH**. Granulosa cells isolated from immature DES-primed rats were cultured and treated with FSH (30 ng/ml) for the indicated times, and the total RNA or protein was extracted. A: RT-PCR was performed with the specific primer sets for POR, aromatase, and GAPDH. B: Results of real-time PCR are also shown. Data are the mean ± SEM values of at least three independent experiments. *, *P *< 0.01. C: Western blot analysis was performed with antibodies to POR, aromatase, and GAPDH, using the same lysates.

POR mRNA and proteins were strongly induced by FSH in granulosa cells after 3–6 h. They fell temporarily at 12 h, and then increased again from 24–48 h (Fig. 3A, B and 3C). Cyp19 mRNA and protein were barely detectable before FSH treatment. They were strongly induced by FSH, and followed a similar pattern to that of POR, although Cyp19 levels responded more quickly than POR levels. Consistent with the results from MSCs, POR was induced in the ovary and granulosa cells when they were stimulated to differentiate into steroidogenic cells by gonadotropins. Induction of Cyp19 protein by FSH causes ovarian estrogen synthesis and follicle maturation [26]. These observations suggest that FSH-induced up-regulation of POR expression, as well as that of Cyp19, could enhance estrogen synthesis in granulosa cells.

### Effects of POR expression on estrogen synthesis

**Figure 4:**
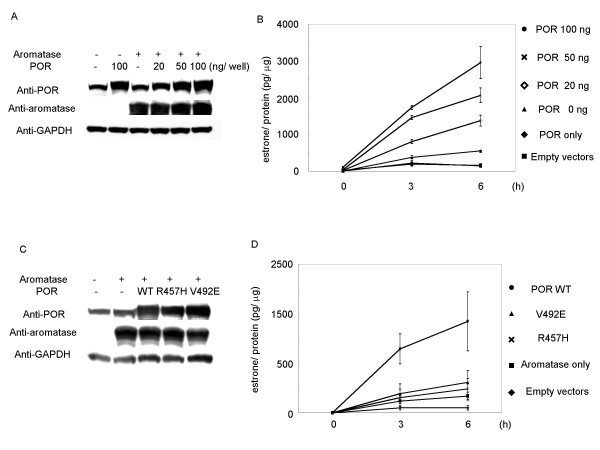
**Production of estrone by co-transfection of wild-type or mutant POR and aromatase expression vectors in COS-7 cells**. A and B: Amounts of estrone in the culture medium were detected by EIA at the indicated times and for the indicated contents of POR expression vector, with or without aromatase expression vector. The protein expression of POR, aromatase, and GAPDH after transfection with these amounts of vectors was detected by western blot analysis. C and D: COS-7 cells were transfected with wild-type POR or R457H or V492E mutant expression vectors (20 ng/well) with aromatase expression vector, at the indicated times. Data are means and SEM values (B) or SD values (D) of at least three assays, respectively.

In order to determine if estrogen synthesis was affected by the level of POR expression when aromatase expression remained constant, POR and aromatase expression vectors were transiently co-transfected into COS-7 cells that expressed an undetectable level of aromatase protein (identified by Western blotting) and low levels of POR expression (Fig. 4A and 4B). In order to investigate aromatase enzyme activity, we measured the conversion of androstenedione to estrone in the culture medium. Estrone levels were low and were unaffected by co-transfection with both control vectors, or with the POR expression vector alone. However, time-dependent accumulation of estrone occurred when aromatase was expressed, and estrone levels were further increased by co-transfection with the POR expression vector. Co-transfection with the aromatase expression vector and 100 ng of the POR expression vector resulted in a 6-fold increase in the amount of estrone converted in 6 h, compared with the aromatase vector alone (Fig. 4A and 4B). These results suggest that estrogen synthesis is up-regulated by increasing levels of POR expression, even when aromatase expression is constant.

We then examined the effects of the expression of mutant POR genes on estrone production. It has been reported that aromatase activity was completely lost when the R457H or V492E POR mutant proteins were expressed instead of the wild-type protein [27]. In COS-7 cells co-transfected with R457H or V492E mutant POR and aromatase expression vectors, the amount of estrone was reduced to 25% compared to co-transfection with wild-type POR and aromatase. These estrone levels were similar to those seen when aromatase was expressed alone (Fig. 4C and 4D). These results suggest that estrogen synthesis is dependent not only on the expression levels of aromatase protein, but also on the expression levels of POR protein.

### Effects of knockdown of endogenous POR protein on estrogen production

**Figure 5:**
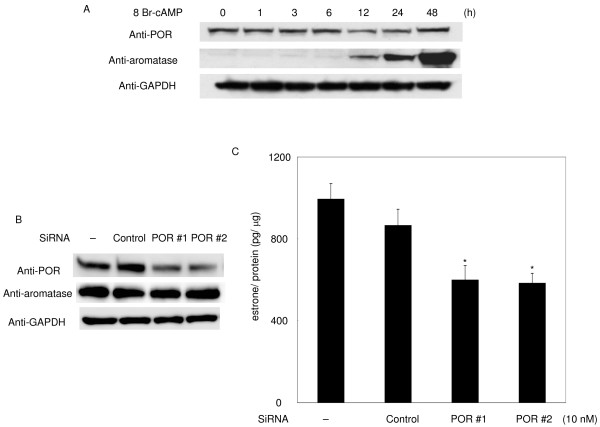
**Effect of POR knockdown on estrone production and cAMP response of POR and aromatase genes in KGN cells**. After transfection with the indicated siRNAs, the culture medium was replaced with that containing 1 mM 8-Br-cAMP and 10 μM androstenedione, and the amount of estrone in the culture medium was detected by EIA 48 h later (B and C). Data are means and SEM values of at least three assays. *, Significant difference in estrone production between KGN cells and KGN cells transfected siRNA for POR#1 or #2 (*P *< 0.01). The protein expression under these culture conditions at the indicated times is also shown (A and B).

We investigated the effects of knockdown of endogenous POR by siRNA in granulosa cell-derived KGN cells. Androstenedione and 8-Br-cAMP were added to the culture medium 24 h after transfection with 10 nM siRNA and the amount of estrone in the medium was measured after 48 h. In contrast to the rat granulosa cells, POR expression was relatively high in KGN cells and was not greatly changed by 8-Br-cAMP. Aromatase expression, however, was induced by 8-Br-cAMP, as in rat granulosa cells treated with FSH (Fig. 5A). POR siRNA caused a reduction in the amount of estrone to about 70% of that in cells transfected with a scrambled control siRNA (Fig. 5C). No estrone was detected in the absence of 8-Br-cAMP.

## Discussion

In our previous study, we reported that stable transfection of MSCs with SF-1, followed by treatment with cAMP, led to their conversion into steroidogenic cells [16]. These cells offer a possible clinical source of stem cells for the treatment of diseases of steroidogenic organs, and represent a powerful tool for studying the differentiation of the steroidogenic lineage [16,28-30]. In this study, using DNA microarray, RT-PCR, and real-time PCR analysis, we observed that POR mRNA was induced during the differentiation of hMSCs into steroidogenic cells. We also demonstrated that the expression of the POR mRNA and protein were up-regulated by FSH during the differentiation of rat granulosa cells into steroidogenic cells. Similar phenomena have been observed in adrenocortical cell lines stimulated with adrenocorticotropic hormone (ACTH) or cAMP [31,32]. These results suggest that POR expression in steroidogenic cells is regulated by the pituitary hormone/cAMP pathway. In contrast, it has been reported that POR gene expression in hepatic cells and other organs was not changed by any stimuli, including cAMP, and in support of these observations, the promoter region of the POR gene supported constitutively high luciferase activity that was unaffected by any stimuli [33-36]. These results suggest that regulation of POR gene expression differs between steroidogenic and non-steroidogenic cells. Although the reasons for this difference remain unclear, the expression of SF-1 in steroidogenic cells may explain this phenomenon. SF-1 is expressed in steroidogenic cells such as adrenocortical cells, testicular Leydig cells, ovarian theca and granulosa cells, and regulates steroidogenic genes that encode cytochrome P450scc, StAR and CYP19, by binding to cAMP-response sequences [3,22,23]. Our results from MSCs indicate that the POR gene could represent a new target for SF-1 in steroidogenic cells. It is therefore conceivable that the expression of POR is regulated by the pituitary hormone/cAMP pathway. Such regulations may also cause similar fluctuations in POR and CYP19 expression in the ovary and in granulosa cells. However, CYP19 mRNA was induced more rapidly than POR mRNA, because the CYP19 gene is regulated by CREB, as well as by SF-1 [3]. On the other hand, our preliminary study indicated that CREB was not involved in the induction of POR mRNA in granulosa cells by FSH (data not shown). The POR gene spans more than 70 kb, including an untranslated first exon residing about 38.8 kb upstream of the coding region [4]. Further studies are needed to understand the mechanisms regulating POR gene expression.

Based on our observations in rat ovary and in granulosa cells stimulated by gonadotropins, it is likely that increasing POR expression induced by gonadotropins further up-regulates aromatase activity, and that estrogen production is regulated not only by aromatase expression, but also by POR. This proposition is supported by the augmentation of aromatase activity coincident with increasing POR expression in COS-7 cells, despite a constant expression of aromatase. Hall and his colleagues, using purified enzymes, also reported that the activity of CYP17 was increased by the addition of POR protein in a dose-dependent manner, resulting in increased androgen production [37]. Because almost all steroidogenic enzymes are up-regulated by the pituitary hormone/cAMP pathway, our model should be applicable to other microsomal steroidogenic P450s, such as CYP17 and CYP21. In support of this hypothesis, CYP17 expression was markedly increased, together with POR, in SF-1-transduced MSCs. Similarly, the expression of ferredoxin, which is a member of the electron transport chain in mitochondria and regulates pregnenolone production by P450scc, is known to be increased by pituitary hormones and cAMP [38-40]. Therefore, it is possible that, in addition to steroidogenic enzyme expression, up-regulation of electron transporter expression plays a vital role in steroid hormone production by gonadotropins and ACTH. This mechanism should be conserved between mitochondrial and microsomal steroidogenesis.

In COS-7 cells, a low level of estrone was detected in the culture medium in the absence of transfection of CYP19, even though aromatase proteins were not detected by western blotting. COS cells (COS-1 and COS-7) have often been used for measuring aromatase activity by ectopic expression of the CYP19 gene, and low aromatase activities have been reported under non-transfected conditions [41-44]. It is thus conceivable that aromatase proteins are expressed at very low levels in this cell line. It has been reported that the R457H and V492E POR mutations are associated with loss of P450 enzyme activities, because these mutations in the FAD domain hamper the ability of POR to transfer electrons to its FMN group, which ultimately provides electrons to P450 enzymes [27]. When these mutants were expressed concomitantly with aromatase in COS-7 cells, estrone production was similar to that seen with aromatase alone. This result indicates that the augmentation of aromatase activity by POR is not a side effect of overexpression of POR, but is due to the increased electron supply resulting from increased POR expression in COS-7 cells.

FSH, forskolin, and cAMP have been reported to cause increases in aromatase activity in KGN cells (a human ovarian granulosa-like tumor cell line), as well as in rat granulosa cells [45,46]. In contrast, POR expression in KGN cells was little changed by 8-Br-cAMP treatment, and was much higher than in primary cultures of rat granulosa cells. In addition, it has been reported that the lower amount of POR protein, compared with the amount of P450 protein, was still sufficient to carry out all metabolic reactions [47]. Hence, the amount of POR expression after inhibition by siRNA, still seemed to be sufficient to activate aromatase induced by 8-Br-cAMP. Therefore, estrone production should not be greatly reduced by POR siRNA. POR is involved not only in steroidogenesis, but also in drug and xenobiotic metabolism, and in tumorigenesis [48,49]. It is likely that the hormone-responsiveness of the POR gene is lost during immortalization of KGN cells. However, induction of POR in granulosa cells may play a more significant role in estrogen production, because the amount of POR protein in these cells is limited. In granulosa cells, POR and aromatase expression are regulated by gonadotropins, which in turn may be responsible for coordinated regulation of steroid hormone production at each stage of follicular growth.

In this study, we have demonstrated that estrogen production was caused not only by aromatase expression, but also through the promotion of aromatase activity by FSH-induced POR expression in ovarian granulosa cells. POR knockout mice are embryonically lethal, but POR heterozygous knockout mice are fertile, and do not have ambiguous genitalia [50]. Our study suggests that a comparison of ovarian function and morphology between POR heterozygous knockout mice and aromatase knockout mice [51] could provide further useful information. In addition, POR might be involved in the differentiation of these steroidogenic organs through activation of microsomal P450 enzymes.

## Competing interests

The authors declare that they have no competing interests.

## Authors' contributions

YI performed all experiments with following coauthors helps. TY generated steroidogenic cells from UE7T-13. TM supported the experiments of real-time PCR and western blotting. KK and KK carried out the analysis of DNA microarray. MU supported the primary culture of granulosa cells. AU made UE7T-13 cells. KM conceived of the study, and participated in its design and coordination and helped to draft the manuscript. All authors read and approved the final manuscript.
